# Rare Vascular Anomalies in an Adult: Absent Left Pulmonary Artery With a Right-Sided Aortic Arch

**DOI:** 10.7759/cureus.43020

**Published:** 2023-08-06

**Authors:** Matthew League, John Eick

**Affiliations:** 1 Internal Medicine, Lincoln Memorial University - DeBusk College of Osteopathic Medicine, Harrogate, USA; 2 Internal Medicine, Methodist Le Bonheur Healthcare, Memphis, USA

**Keywords:** congenital heart anomalies, cardiovascular anomaly, right aortic arch, pulmonary artery absence, uapa

## Abstract

Congenital absence of the left pulmonary artery remains a rarely reported anomalous condition and is even less commonly seen in conjunction with a right-sided aortic arch. While most cases are identified during prenatal fetal ultrasonography and require early childhood intervention, some asymptomatic cases can go unrecognized until incidentally detected on chest imaging as an adult. This case details a 31-year-old male with a congenital absence of the left pulmonary artery and right-sided aortic arch with subsequent atretic and fibrotic lung, all found on imaging during admission for acute alcoholic hepatitis.

## Introduction

The unilateral absence of pulmonary artery (UAPA) was first observed by Frantzel in 1868 [1]. UAPA is believed to arise from a malformation of the sixth aortic arch during embryogenesis [2]. It has been estimated that the incidence of UAPA is approximately one in 200,000 to one in 300,000 persons [3]. One recent study showed that the occurrence of left-sided UAPA may slightly predominate compared to right-sided. It is also more common to see the co-occurrence of other congenital heart defects with left-sided UAPA, the most common being atrial septal defect, patent foramen ovale, patent ductus arteriosus, and ventricular septal defect [4]. Other congenital cardiovascular anomalies associated with UAPA include tetralogy of Fallot, coarctation of the aorta, right aortic arch, truncus arteriosus, and pulmonary atresia [3].

Isolated right-sided aortic arch incidence is estimated to be 165 in 1000 persons, with a significant association with DiGeorge syndrome [5]. The majority of people with right-sided aortic arches are asymptomatic, and the anomaly is often found incidentally. While there are known cases of UAPA with a right-sided aortic arch, no studies to our knowledge have been done to estimate the incidence of this co-occurrence [6].

Although many cases of UAPA are asymptomatic, some present with vague constellations of pulmonary symptoms, including recurrent pulmonary infections, hemoptysis, dyspnea, reduced exercise tolerance, chest pain, pulmonary hypertension, and high-altitude pulmonary edema [7]. It is hypothesized that the repeated pulmonary infections observed in UAPA result from minimal blood flow to the affected lung, leading to a diminished immune response and impaired ciliary function [7].

## Case presentation

A 31-year-old male with a past medical history of alcoholic fatty liver disease and alcohol-induced cardiomyopathy presented to the Emergency Department with complaints of nausea, fatigue, sore throat, right lower quadrant abdominal pain, dark-colored stools, and yellowing of the skin and eyes. Otherwise, he had no known additional cardiac history, respiratory conditions, or prior surgeries. On examination, he had normal respiratory effort, diminished lung sounds on the left, and minimal left-sided tracheal deviation. A cardiovascular examination revealed a regular heart rhythm with no significant murmurs, rubs, or gallop. Distal radial and dorsalis pedis pulses were equal and 2+ bilaterally.

**Figure 1:**
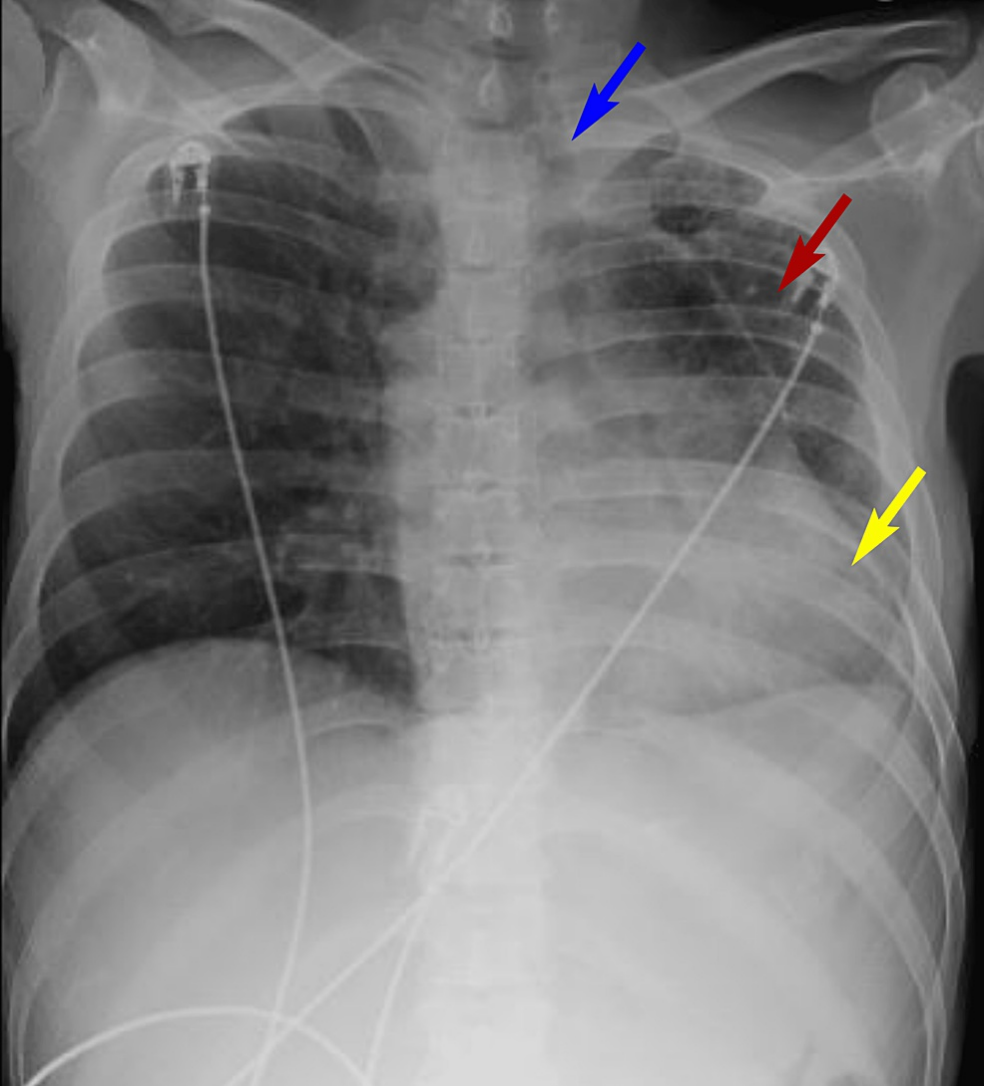
Chest radiograph, posterior-anterior view Chest radiograph posterior-anterior view shows a hypoplastic left lung (red arrow) with right-to-left tracheal deviation (blue arrow) and a prominent cardiac silhouette (yellow arrow) overlying the left thorax.

**Figure 2:**
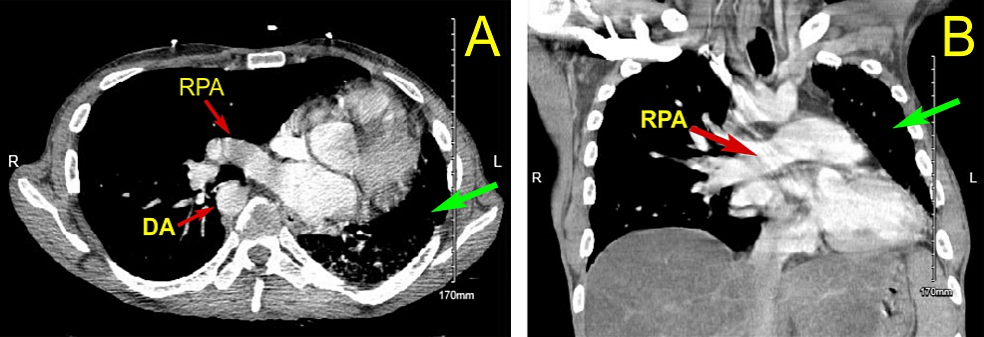
CT thorax with IV contrast, axial and coronal images CT thorax with IV contrast axial image (A), and coronal image (B) demonstrate an absent left pulmonary artery, enlarged right pulmonary artery, and right-sided aortic arch. In addition, the left lung exhibited marked hypoplasia (green arrows). RPA - right pulmonary artery; DA - descending aorta

Chest radiographs were obtained that showed left-sided volume loss with right-to-left tracheal deviation and a prominent cardiac silhouette overlying the left thorax (Figure 1). Radiology recommended obtaining computed tomography (CT) scan of the thorax to further characterize the anomaly (Figure 2). This showed chronic volume loss of the left lung with diffuse chronic interstitial prominence and infiltrate on the left. There was also a shift of the heart and mediastinum to the left, a congenital absence of the left pulmonary artery, and a right-sided aortic arch. The heart was enlarged, and the right lung was compensatorily hyperexpanded and clear. Since the patient had no significant cardiac or respiratory symptoms, no further tests were ordered to investigate these findings during his hospital stay.

The patient was evaluated by hepatology and treated for acute alcoholic hepatitis with a four-week course of prednisolone 40 mg per day. Upon discharge, the patient was referred to outpatient hepatology for continued management of his liver condition. In addition, transthoracic echocardiography was ordered for the patient to complete in an outpatient setting and follow-up with outpatient cardiology.

## Discussion

This case demonstrates the occurrence of a particularly rare cause of chronic volume loss and interstitial fibrotic change in an adult male without respiratory symptoms. CT of the thorax revealed the rare anatomical findings of UAPA with an associated right-sided aortic arch.

The current gold standard of UAPA diagnosis is conventional computed tomography pulmonary angiography (CTA), as this has the advantage of highlighting spatial orientation and extent of peripheral blood vessels. UAPA is also sometimes referred to as unilateral interruption of the pulmonary artery since, classically, on CTA, it will show the beginning of the pulmonary artery branch with a blind end while the distal pulmonary arterial tree is maintained [4,8]. Other nonspecific common radiographic findings of UAPA include volume loss to the ipsilateral lung with overinflation and mediastinal shift. The affected lung will often appear hyperlucent from a reduction in pulmonary blood volume, while a prominent pulmonary artery is seen in the contralateral lung [7].

As it stands, there is currently no agreed-upon consensus for the treatment of UAPA. Management of UAPA revolves around treating any arising clinical symptoms and is dependent upon the potential congenital anomalies associated with it and the presence or absence of pulmonary hypertension. Early identification of UAPA in infancy with transthoracic color-doppler echocardiography and confirmatory chest MRI is crucial to establishing a correct diagnosis [9]. The adult asymptomatic patient should be closely monitored for signs and symptoms of pulmonary hypertension, as this can be a serious complication.

One study by Tian and Zheng highlights the usefulness of echocardiography in the characterization of UAPA and monitoring of pulmonary hypertension, which was found to be a common complication in UAPA patients. In their study, the most common finding associated with a diagnosis of UAPA with pulmonary hypertension was growing collateral arterial supply, likely as compensation for hypoxemia. Over time, increased pulmonary artery pressures can lead to heart failure, which is why pulmonary hypertension leads to a worse prognosis in those with UAPA [4]. The use of echocardiography is an important advancement in the characterization of UAPA and is useful in guiding the treatment of its complications.

## Conclusions

Congenital unilateral absence of the pulmonary artery is a rare cardiovascular malformation that can present with associated cardiovascular anomalies. Isolated unilateral absence of a pulmonary artery can be asymptomatic; however, the congenital anomalies associated with it often require prompt childhood intervention. Early identification and appropriate diagnosis are critical components in the management and care of patients with this rare anomaly, especially those with complications such as pulmonary hypertension. Echocardiography in the characterization of UAPA with pulmonary hypertension appears to be a promising avenue for further study to ensure successful long-term patient outcomes.
